# GDF‐15 is associated with sarcopenia and frailty in acutely admitted older medical patients

**DOI:** 10.1002/jcsm.13513

**Published:** 2024-06-18

**Authors:** Rikke S. Kamper, Hanne Nygaard, Louis Praeger‐Jahnsen, Anette Ekmann, Sisse Bolm Ditlev, Martin Schultz, Sofie Krarup Hansen, Pernille Hansen, Eckart Pressel, Charlotte Suetta

**Affiliations:** ^1^ Department of Geriatric & Palliative Medicine Copenhagen University Hospital, Bispebjerg and Frederiksberg Copenhagen Denmark; ^2^ CopenAge, Copenhagen Center for Clinical Age Research University of Copenhagen Copenhagen Denmark; ^3^ Department of Emergency Medicine Copenhagen University Hospital, Bispebjerg and Frederiksberg Copenhagen Denmark; ^4^ Copenhagen Center for Translational Research Copenhagen University Hospital, Bispebjerg and Frederiksberg Copenhagen Denmark; ^5^ Department of Geriatrics Copenhagen University Hospital, Hvidovre and Amager Hvidovre Denmark; ^6^ Department of Clinical Medicine, Faculty of Health University of Copenhagen Copenhagen Denmark

**Keywords:** Biomarker, Frailty, GDF‐15, Old age, Sarcopenia, Senescence

## Abstract

**Background:**

Growth differentiation factor‐15 (GDF‐15) has been associated with senescence, lower muscle strength, and physical performance in healthy older people. Still, it is not clear whether GDF‐15 can be utilized as a biomarker of sarcopenia and frailty in the early stages of hospitalization. We investigated the association of plasma GDF‐15 with sarcopenia and frailty in older, acutely admitted medical patients.

**Methods:**

The present study is based on secondary analyses of cross‐sectional data from the Copenhagen PROTECT study, a prospective cohort study including 1071 patients ≥65 years of age admitted to the acute medical ward at Copenhagen University Hospital, Bispebjerg, Denmark. Muscle strength was assessed using handgrip strength, and lean mass was assessed using direct segmental multifrequency bioelectrical impedance analyses and used to clarify the potential presence of sarcopenia defined according to guidelines from the European Working Group on Sarcopenia in Older People. Frailty was evaluated using the Clinical Frailty Scale. Plasma GDF‐15 was measured using electrochemiluminescence assays from Meso Scale Discovery (MSD, Rockville, MD, USA).

**Results:**

We included 1036 patients with completed blood samples (mean age 78.9 ± 7.8 years, 53% female). The median concentration of GDF‐15 was 2669.3 pg/mL. Systemic GDF‐15 was significantly higher in patients with either sarcopenia (*P* < 0.01) or frailty (*P* < 0.001) compared with patients without the conditions. Optimum cut‐off points of GDF‐15 relating to sarcopenia and frailty were 1541 and 2166 pg/mL, respectively.

**Conclusions:**

Systemic GDF‐15 was higher in acutely admitted older medical patients with sarcopenia and frailty compared with patients without. The present study defined the optimum cut‐off for GDF‐15, related to the presence of sarcopenia and frailty, respectively. When elevated above the derived cutoffs, GDF‐15 was strongly associated with frailty and sarcopenia in both crude and fully adjusted models.

## Introduction

Ageing at the cellular level, termed senescence, has been described as a cell cycle arrest with accompanying phenotypic and functional alterations,^1^, ^2^ which increase with age^3^ and age‐related diseases.^4^ Although senescence depends on both the type of cell and the inducer of senescence, a recent study has shown that growth differentiation factor‐15 (GDF‐15) is one of the shared features of senescent cells cultivated in the laboratory and in the plasma of the ageing population.^5^ GDF‐15 is a pleiotropic cytokine expressed in multiple tissues throughout the body and has been implicated in various pathological conditions such as metabolic disorders, cardiovascular disease, cancer, and chronic kidney disease.^6^ As with the myokine interleukin‐6 (IL‐6), circulating GDF‐15 is upregulated by bouts of exercise.^7^, ^8^ However, this upregulation is transient and represents a normal physiological response of the muscle tissue being metabolically challenged.^9^ This is supported by the fact that active individuals have lower resting levels of GDF‐15 compared with sedentary individuals.^8^ Notably, although senescence plays a key role in wound repair, embryogenesis, and development processes,^10^, ^11^, ^12^ chronically elevated levels of systemic senescence as observed with ageing or various disease states is likely detrimental.^9^

We have previously reported increases in systemic GDF‐15 with age in healthy men and women and a negative relationship between circulating GDF‐15 levels and relative muscle power,^13^ supporting the evidence of increased senescence with age and a potential effect on muscle performance. This is in line with other studies of healthy adults demonstrating that higher levels of GDF‐15 are associated with lower muscle strength, lower physical performance, and slower gait‐speed^14^, ^15^ and that GDF‐15 levels are lower in individuals with an active lifestyle.^8^ Notably, although higher GDF‐15 levels were associated with lower muscle strength and lower muscle mass in older women, GDF‐15 levels could not discriminate between non‐sarcopenic and sarcopenic individuals.^16^

Due to the association of GDF‐15 with various age‐related pathologies, it has also been proposed as a core candidate biomarker of frailty,^17^ yet more research is needed on the subject. To the best of our knowledge, only one previous study has investigated the association of GDF‐15 with frailty and age‐related physical decline in acutely admitted older patients.^18^ Whether plasma GDF‐15 can be utilized as a biomarker in this population is yet to be fully elucidated due to its concurrent association with many chronic age‐related diseases. Furthermore, the gene expression of GDF‐15 may be affected by ongoing inflammation and infection.^9^ As such, it is unclear whether GDF‐15 can be utilized as a biomarker of sarcopenia and frailty in the early stages of hospitalization due to the potential effects of acute illness. We aim to investigate the association of plasma GDF‐15, measured within the first 24 h of an acute admission, with sarcopenia and frailty in older medical patients.

## Methods

### Subjects and study design

This study is based on secondary analyses of cross‐sectional data from the Copenhagen PROTECT study, a prospective cohort study investigating biomarkers of prolonged hospitalization, readmission, and mortality, registered at Clinicaltrials.gov (NCT04151108). The present work includes 1036 acutely admitted older medical patients ≥ 65 years of age admitted to Copenhagen University Hospital, Bispebjerg & Frederiksberg, between November 2019 and November 2021 with completed blood samples. Patients were excluded from participation due to; admission exceeding 24 h prior to baseline assessment, terminal illness evaluated by data from the healthcare journal in close collaboration with the treating doctor, inability to read or speak Danish, airborne or droplet infections requiring isolation (none of the enrolled patients were hospitalized due to SARS‐COV‐2), medical contraindications for participation evaluated by health personnel, or temporary civil registration number.^19^ All assessments were performed within 24 h of admission as per the protocol.^19^ Written informed consent was obtained from all enrolled patients or from relatives in collaboration with a doctor not related to the project in cases where the patients could not provide consent due to persistent or fluctuating cognitive impairment. The study was performed in accordance with the Declaration of Helsinki and approved by the local ethics committee of Copenhagen and Frederiksberg.

### Sarcopenia and frailty

Sarcopenia was evaluated in patients with complete records of body composition and muscle strength and was evident when both low muscle mass and low muscle strength were present. Muscle mass was assessed in patients with complete measurements of body composition (*n* = 631) using direct segmental multifrequency bioelectrical impedance analyses (DSM‐BIA) (Inbody S10; BioSpace Co., Ltd, Seoul, South Korea) and reported as appendicular lean mass adjusted for height^2^ denoted here as the skeletal muscle index (SMI). Low muscle mass was defined as an SMI below <7.0 kg/m^2^ and <5.5 kg/m^2^ for men and women, respectively, as recommended by the recent guidelines from the EWGSOP.^20^ Muscle strength was assessed as handgrip strength (HGS) using a digital hand‐held dynamometer (Model SH1001; SAEHAN Corporation, Yangdeok‐Dong, Masan, South Korea). The highest value of three attempts with the dominant hand was denoted as the result, as per the protocol.^19^ Low HGS was defined as a HGS below <27 kg and <16 kg for men and women, respectively, as proposed by the recent guidelines from the European Working Group on Sarcopenia in Older People (EWGSOP).^20^ Frailty was evaluated using the 9‐point Clinical Frailty Scale (CFS) with frailty evident at scores ≥5.^21^

### Patient‐reported data

Cognitive impairment was evaluated using the Orientation, Memory, and Concentration test, which ranged from 0 to 28.^22^ Malnutrition was assessed using the Short Nutritional Assessment Questionnaire (SNAQ), which ranged from 0 to 5.^23^

### Growth differentiation factor‐15

Blood samples were drawn from the antecubital vein within 24 h of hospitalization. Blood was collected in EDTA‐treated tubes and kept on ice with subsequent centrifugation at 2500 g at 4°C for 10 min. Plasma was then extracted and kept at −80°C until analyses.

Plasma was analysed for GDF‐15 using R‐PLEX electrochemiluminescence assays from MesoScale Diagnostics (MSD, Rockville, MD, USA). Samples were analysed according to the protocol provided by MesoScale, with a few alterations.

The samples were analysed in three separate batches. The first 20 samples were analysed as duplicates at four different dilutions to determine the optimal dilution factor that would provide the most suitable concentration range for subsequent measurements. The four dilutions were 10‐fold, 50‐fold, 100‐fold, and 150‐fold. Based on the results from the first batch, the optimal dilution factor was established as a 150‐fold dilution. This dilution factor was selected as it likely provided the most reliable and consistent measurement range for the subsequent analyses. In the second batch, 124 samples (10% of the total number of samples) were analysed as duplicates using the established 150‐fold dilution factor. This procedure was performed to ensure the reproducibility and precision of the measurements at the optimal dilution. The intra‐assay coefficient of variance (CV) for the duplicate samples in this batch was calculated to be 2.0%, indicating a high level of precision and consistency in the measurements. As such, the final batch of samples was analysed as singlets using the following protocol.

Reagent dilutions and reagents, including GDF‐15 capture and detection antibodies, assay buffers, and calibration standards, were brought to room temperature. Capture antibodies were immobilized on the plates for 1 h at room temperature at 800 rpm shaking. Subsequently, the plates were washed three times with PBS + 0.05% Tween20. The stock calibrator was dissolved in assay buffer and incubated for 30 min at room temperature prior to dilution. Plasma samples were diluted to the desired dilutions and added to the wells next to the calibration standards. Plates were left to incubate for an hour at room temperature with 800 rpm shaking and subsequently washed three times. The detection antibody was added to the plates and incubated for 1 h. The plates were then washed to remove any excess detection‐antibodies. The electrochemiluminescent detection reagent was added to the wells, and the emission signal from each well was measured using the Meso QuickPlex SQ 120 instrument and analysed using Discovery Workbench, version 4.0.12.

### Information from medical records

Information regarding medical diagnoses was extracted from hospital medical records, and co‐morbidity was classified according to the age‐adjusted Charlson co‐morbidity index (CCI).^24^ Patients were categorized by the CCI weighted index as having either mild, moderate, or severe co‐morbidity represented by CCI scores of 1–2, 3–4, or ≥5, respectively. The presence of polypharmacy was assessed by counting all prescribed medication upon acute admission, with a number of medications > 5 indicating the presence of polypharmacy, as described in detail elsewhere.^19^ The Early Warning Score (EWS) upon admission was extracted from the medical records, where the total score is based on routinely measured physiological parameters (respiratory rate, heart rate, blood pressure, oxygen saturation, temperature, and a simple assessment of consciousness) to identify a physiological deterioration in patients acutely admitted to the hospital.^25^ Data from clinical bloodwork performed in the initial 24 h of hospitalization included C‐reactive protein (CRP) and glomerular filtration rate (e‐GFR). Residential dependency was defined as either living in own home or nursing home.

### Statistics

Kolmogorov–Smirnov analyses and visual inspection were used to evaluate the normality of distributions. Descriptive statistics were performed to evaluate relative percentages and mean or median values depending on the distribution. Patients' characteristics are presented as a total, followed by differences between the frail and non‐frail groups. Moreover, a total presentation of the subgroup of patients assessed for sarcopenia is given, followed by a presentation of differences in the characteristics between the sarcopenic and non‐sarcopenic groups. Youden's Index^26^ was calculated to establish the optimum cutoff value for GDF‐15 for frailty and sarcopenia, respectively.

The dichotomized GDF‐15, presenting values under or over the established cutoff, were assessed in a multivariable logistic regression model to investigate the association of GDF‐15 with frailty and sarcopenia, respectively. The results were presented as a crude model, as a fully adjusted model with all confounders found to be significantly different between groups, and lastly, a final model established using backward elimination. In the backward elimination, one variable at the time was deleted, starting with the least significant. This procedure continued until all variables included in the models were significantly associated with the outcome. Additionally, we tested interactions between GDF‐15 and all covariates. Regarding sarcopenia, we found an interaction between GDF‐15 and the CCI. Thus, a stratified grouping on the different levels in the weighted index of the CCI was performed in relation to the dichotomized GDF‐15. For frailty, we found an interaction between GDF‐15 and both the CCI and sex, and have performed stratified analysis on these confounders, including sex‐related risk differences.

The pictorial display of the differences in GDF‐15 levels across groups of sarcopenic/non‐sarcopenic and frail/non‐frail, were analysed using univariate general linear models with Bonferroni corrections and with subsequent adjustments for the effect of age, sex, co‐morbidity, and C‐reactive protein. The significance level was set at *P* < 0.05. Analyses were performed using IBM SPSS Statistics, Version 29.01.0.

## Results

This study used data from acutely admitted older patients enrolled in the Copenhagen PROTECT study with completed blood samples (*n* = 1036). Patient characteristics are presented in Table 1. Patients had a mean age of 78.9 years; 53% were female. The median concentration of GDF‐15 was 2669.2 pg/mL in the total cohort and 2365.5 pg/mL in the subgroup of patients assessed for potential sarcopenia. Frail patients had a significantly higher concentration of GDF‐15 compared with non‐frail, and the corresponding analysis in the subgroup revealed a significantly higher concentration of GDF‐15 in the sarcopenic patients compared with the non‐sarcopenic (Table 1).

**Table 1:** Characteristics of acutely admitted older patients presented as the total cohort, frail patients, and a subgroup of patients assessed for potential sarcopenia.

	Total (*n* = 1036)	Frail patients (*n* = 502)	*P*‐value	Subgroup (*n* = 628)	Sarcopenic patients (*n* = 126)	*P*‐value
Age (years)	78.9 ± 7.8	80.7 ± 8.1	*P* < 0.001	78.2 ± 5.6	81.0 ± 8.4	*P* < 0.001
Female (%)	53.0	56.8	*P* < 0.02	55.1	50.8	*P* > 0.2
GDF‐15^a^ (pg/mL)	2669.2 (1538.0;4472.7)	3048.5 (1793.2;5103.8)	*P* < 0.001	2365.5 (1335.7;3971.5)	2729.2 (1679.1;4909.6)	*P* < 0.001
Comorbidity Index (%)			*P* < 0.001			*P* < 0.02
Mild comorbidity	3.3	1.8		4.0	3.2	
Moderate comorbidity	38.6	28.5		42.0	31.0	
Severe comorbidity	58.1	69.7		54.0	65.9	
Medication (number)	8.5 ± 4.4	9.7 ± 4.3	*P* < 0.001	8.1 ± 4.4	8.5 ± 4.5	*P* > 0.2
Polypharmacy (%)	72.8	81.3	*P* < 0.001	70.5	71.4	*P* > 0.2
Early Warning Score^a^	2 (0;4)	2 (0;4)	*P* > 0.2	2 (0;4)	1 (0;3)	*P* > 0.5
CRP^a^	27 (5;98)	21 (4;76)	*P* < 0.02	23 (4;99)	31 (4;89)	*P* > 0.6
eGFR (mL/min/1.73 m^2^)^a^	66 (46;83)	62 (43;83)	*P* < 0.01	70 (49;84)	67 (45;84)	*P* > 0.4
Low HGS^b^ (%)	46.8	63.8	*P* < 0.001	39.5	50.8	*P* < 0.001
Low SMI^c^ (%)	32.5	42.7	*P* < 0.001	32.6	61.5	*P* < 0.001
Sarcopenia^d^ (%)	20.0	31.6	*P* < 0.001	‐	‐	
Frailty (%)	48.4	‐	‐	38.9	61.1	*P* < 0.001
BMI^e^ (kg/m^2^)	25.8 ± 5.3	25.0 ± 5.5	*P* < 0.03	25.7 ± 5.2	21.9 ± 3.5	*P* < 0.001
SNAQ (score)^f^	1 (0;3)	1 (0;4)	*P* < 0.03	1 (0;3)	1 (0;4)	*P* < 0.01
Cognitive impairment, OMC (score)^a^ ^,^ ^g^	22 (17;26)	20 (15;24)	*P* < 0.001	23 (18;26)	19.5 (14;24)	*P* < 0.001
Medical specialties (%)			*P* < 0.001			*P* < 0.01
Geriatric medicine	37.5	45.1		65.4	50.0	
Lung‐ and infectious medicine	30.7	28.1		34.6	27.8	
Endocrinology	20.8	18.0		20.7	14.3	
Gastroenterology	10.6	8.4		9.7	7.9	
Acute medicine	0.3	0.4		0.0	0.0	
Residential dependency (%)			*P* < 0.001			*P* < 0.001
Living in own home	92.1	84.1		95.7	88.1	
Nursing home	7.9	15.9		4.3	11.9	

Values are expressed as means ± SD or as relative percentage unless otherwise stated.

BMI, body mass index; CRP,C‐ reactive protein; eGFR, estimated glomerular filtration rate; GDF‐15, growth differentiation factor 15; HGS, handgrip strength; OMC, Orientation Memory Concentration test; SMI, skeletal muscle index; SNAQ, Short Nutritional Assessment Questionnaire.

^a^ Median (IQR).

^b^ *n* = 999.

^c^ *n* = 631.

^d^ *n* = 628.

^e^ *n* = 724.

^f^ *n* = 644.

^g^ *n* = 947.

As displayed in Table 1, the frail and the sarcopenic patients had a significantly higher proportion of severe co‐morbidity, lower cognitive scores on the OMC, a lower BMI, and higher SNAQ scores compared with their respective reference groups. Regarding the number of medications, the mean number of medications prescribed in the system was 8.5 in the total cohort, and the proportion of polypharmacy was 72.8%. In frail patients, both the number of medications and the proportion of polypharmacy were significantly elevated compared with the non‐frail. The median EWS was 2, with no significant differences between groups (Table 1). In terms of eGFR and CRP, both biomarkers were significantly lower in the frail group compared with the non‐frail group. No significant differences were found between the sarcopenic and non‐sarcopenic patients (Table 1).

**Figure 1:**
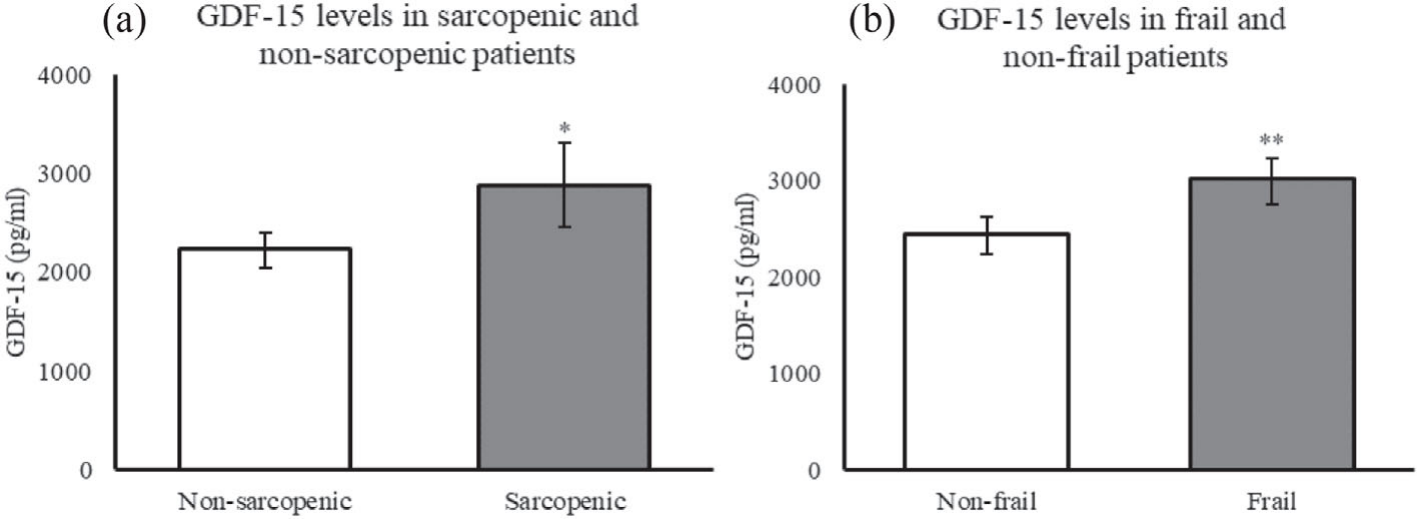
Levels of GDF‐15 according to the presence of a. sarcopenia and b. frailty. They are displayed as the geometric mean with a corresponding 95% CI of the mean. Estimated geometric means are adjusted for age, sex, co‐morbidity, and levels of C‐reactive protein. Growth differentiation factor 15 (GDF‐15). *P < 0.01. **P < 0.001.

Figure 1 displays the difference in levels of GDF‐15 in patients with sarcopenia and frailty compared with patients without these syndromes. GDF‐15 was significantly higher in patients with sarcopenia (*P* < 0.01) (Figure 1A) and patients with frailty (*P* < 0.001) (Figure 1B) compared with non‐sarcopenic and non‐frail patients, respectively. Associations remained significant after adjusting for the effect of age, sex, co‐morbidity, and levels of CRP.

The optimum cutoff of GDF‐15, estimated by Youden's Index, was 2166 and 1541 pg/mL for frailty and sarcopenia, respectively. When dichotomized according to the outcome‐related cutoffs, a total of *n* = 638 had a GDF‐15 level above 2166 pg/mL, and *n* = 778 had a GDF‐15 concentration above 1541 pg/mL in the total group. After applying the outcome‐related cutoffs and investigating the association of dichotomized GDF‐15 with sarcopenia and frailty, respectively, GDF‐15 was found to be associated with the outcomes both in the crude models and the final adjusted models. Results regarding the association between GDF‐15 and sarcopenia, demonstrated an Odds Ratio (OR) (95% CI) in the crude model of 2.3 (1.4–3.7), and an adjusted OR (aOR) (95% CI) of 2.0 (1.2–3.3) in the adjusted final model (Table 2). Correspondingly, results regarding the association between GDF‐15 and frailty demonstrated an OR (95% CI) of 1.8 (1.4–2.3) in the crude model and an aOR (95% CI) of 1.7 (1.3–2.2) in the final model (Table 2).

**Table 2:** Odds ratios for having sarcopenia or frailty according to the dichotomous level of GDF‐15.

	Sarcopenia	Sarcopenia	Sarcopenia	Frailty	Frailty	Frailty
	OR (95% CI), Crude model	OR (95% CI), Model 1^a^	OR (95% CI), Model 2^b^	OR (95% CI), Crude model	OR (95% CI), Model 1^c^	OR (95% CI), Model 2^d^
GDF‐15 Cut‐off: 1541 pg/mL	2.3 (1.4–3.7)	2.0 (1.2–3.3)	2.0 (1.2–3.3)	‐	‐	‐
GDF‐15 Cut‐off: 2166 pg/mL	‐	‐	‐	1.8 (1.4–2.3)	1.7 (1.3–2.3)	1.7 (1.3–2.2)

CCI, Charlson co‐morbidity index; CI, confidence interval; CPR, C‐reactive protein; eGFR, estimated glomerular filtration rate; GDF‐15, growth differentiating factor 15; OR, odds ratio.

^a^ Model adjusted for age, gender, CCI‐groups, and CRP.

^b^ Model adjusted for age.

^c^ Model adjusted for sex, CCI‐groups, age, polypharmacy, CRP, and eGFR.

^d^ Model adjusted for age, polypharmacy, and CRP.

The cases and proportions of GDF‐15 >/< the outcome‐related cutoffs are presented in Table S1. In the sub‐group of patients assessed for potential sarcopenia, we found a significantly higher proportion of patients with GDF‐15 > 1541 pg/mL in the moderate and severe CCI weighted index (*P* > 0.001). When applying the frailty‐related GDF‐15 cutoff in the total group, we identified a significantly higher proportion of patients in the moderate and severe CCI weighted index (*P* > 0.001). Moreover, when stratified by sex related to the GDF‐15 > 2166 pg/mL, the risk difference (95% CI) of the male sex was 0.14 (0.08–0.20) (Table S1).

## Discussion

The literature regarding the association of GDF‐15 with sarcopenia and frailty in older hospitalized patients is scarce. Furthermore, due to the potential effects of acute illness, it is unclear whether plasma GDF‐15 can be utilized as a biomarker of these syndromes when assessed in the early stages of hospitalization. In this study, we investigated the association of GDF‐15 with sarcopenia and frailty in older, acutely admitted medical patients.

The present data demonstrated that acutely admitted patients with sarcopenia had significantly higher levels of systemic GDF‐15 compared with patients without sarcopenia (*P* < 0.01) (Figure 1A). This is in line with recent results from a study on stable COPD patients, which found that serum GDF‐15 levels were significantly higher in patients with sarcopenia compared with non‐sarcopenic patients. Furthermore, systemic GDF‐15 levels could predict the presence of sarcopenia in a validation cohort with good prognostic accuracy.^27^ Our results remained significant after adjusting for the effect of age, sex, co‐morbidity, and levels of CRP, indicating that GDF‐15 may be a potential biomarker of sarcopenia in multimorbid acutely admitted older patients. In contrast, another study found no difference in GDF‐15 levels between non‐sarcopenic and sarcopenic healthy older women.^16^ Notably, the latter study included a small number of healthy subjects and was not based on the recent guidelines and cutoff values for muscle strength and muscle mass from the EWGSOP, which may explain the discrepancies in results.

Frailty is an age‐related condition characterized by increased vulnerability and susceptibility to stressors, increasing the risk of mortality and dependency.^21^, ^28^ To the best of our knowledge, this is the first study to evaluate the association between systemic levels of GDF‐15 and the presence of frailty determined using the Clinical Frailty Scale. Notably, the Clinical Frailty Scale is a screening tool evaluating the level of frailty 2 weeks prior to hospitalization. We found that older acutely admitted patients defined as frail had significantly higher levels of GDF‐15 compared with non‐frail patients, which persisted even after controlling for the effects of age, sex, co‐morbidities, and levels of CRP (Figure 1B). These results indicate that levels of plasma GDF‐15 may serve as a future biomarker of frailty in acutely admitted older patients. Indeed, a comprehensive review evaluated potential biomarkers for frailty and found GDF‐15 to be a core candidate.^17^ Other studies have shown that patients with physical frailty, evaluated by either Fried's Frailty Phenotype or as impairments in activities of daily living (ADL), also demonstrate higher levels of GDF‐15.^29^, ^30^, ^31^

Due to the association of GDF‐15 with both sarcopenia and frailty, it may serve as a pragmatic stratification tool for evaluating older patients at risk. Yet, systemic levels of GDF‐15 may reflect underlying conditions or pathologies in acutely admitted sarcopenic or frail patients. Nevertheless, the mechanisms by which higher levels of GDF‐15 are associated with sarcopenia and frailty are yet to be elucidated. Senescence, however, could be a mechanism underlying the pathology of these geriatric conditions. As previously mentioned, senescence is characterized by cell cycle arrest with accompanying phenotypic and functional alterations.^1^, ^2^ Senescent cells are associated with a distinctive secretory phenotype termed the senescence‐associated secretory phenotype (SASP), consisting of a panel of inflammatory proteins,^32^ and a large production of mitochondrial derived reactive oxygen species (ROS).^33^

Mitochondrial dysfunction has been suggested as a core element of senescence, with the SASP and ROS working in conjunction to reinforce senescence in both an autocrine and paracrine manner.^34^ Studies have shown that although mitochondrial mass may be increased in senescent cells, the mitochondria are less functional, potentially due to decreased mitophagy.^34^ As mitochondria are essential for regulating the metabolic state of muscle tissue, it is not surprising that mitochondrial dysfunction is recognized as a key player in muscle atrophy.^35^ As such, the associations between GDF‐15 and sarcopenia observed in the current study, may be the result of senescence and mitochondrial dysfunction. This could ultimately lead to muscle dysfunction through reduced ATP production, increased mitochondrial ROS production, impaired mitochondrial biogenesis and mitophagy, and decreased mitochondrial quality.^35^ This is in line with a previous study, showing that sarcopenic patients demonstrated altered transcriptional factors in skeletal muscle biopsies, which indicated the presence of mitochondrial dysfunction.^36^ Furthermore, another study found that pre‐existing mitochondrial dysfunction contributed to loss of muscle mass following aortic surgery and that mitochondrial dysfunction was associated with systemic levels of GDF‐15.^37^

The role of senescence in the pathology of frailty becomes somewhat more complex. Frailty, a condition affecting multiple physiological systems, may show distinct associations with senescence depending on the investigated frailty domain. Nonetheless, the age‐related impairment of the immune system concurrent with exponential growth in the senescence burden provides a strong rationale for senescence being a key player in the development of increased vulnerability in frail older individuals.^38^ To the best of our knowledge, this is the first study demonstrating that systemic GDF‐15 is associated with frailty evaluated using the Clinical Frailty Scale. Whether this association is mediated by underlying physical frailty, cognitive frailty, or impairments across multiple physiological systems causing frailty is yet to be investigated. In the analysis of the association between elevated GDF‐15 and frailty, an interaction with sex was significant. Results demonstrated a significantly higher proportion of men with elevated GFD‐15 concentrations compared with women. As such, we estimated a significant positive risk difference related to the male sex. These findings are in accordance with findings stated in a paper by Herpich et al.,^39^ identifying that older male patients had significantly higher levels of GDF‐15 compared with female patients. A prior study by Sun et al.^40^ has identified and discussed the associations between male sex hormones such as testosterone and GDF‐15, but the underlying mechanisms remain unclear.

### Limitations

Notably, systemically measured senescence may not reflect senescence acting at local cellular levels. Furthermore, regarding associations between low muscle strength and sarcopenia, it should be considered that the relative contribution of muscle tissue to the overall systemic senescence may differ from other tissues. Notably, blood samples were extracted within 24 h of acute admission, and the potential effects of acute illness may exacerbate levels of GDF‐15. However, as we wanted to test the hypothesis that GDF‐15 could also be a suitable biomarker in the early stages of acute hospitalization, we controlled for the potential effect of acute illness by adjusting for levels of CRP in statistical analyses. Notably, we do not have data regarding the early use of anti‐inflammatory or steroid drugs upon arrival to the acute ward, which could affect biomarker concentrations. Moreover, we do not have information regarding potential dehydration or fluid overload, which are known conditions to affect the result from the BIA assessment. Lastly, due to the cross‐sectional design of the study, it is not possible to establish causal effects.

### Clinical implications

We found strong associations of dichotomized GDF‐15 with frailty and sarcopenia, respectively, highlighting the clinical usability of our established GDF‐15 cut‐offs. It also emphasizes the importance of continuing the research on the clinical usability and implementation of GDF‐15 as a marker of risk in older acutely admitted patients. Nevertheless, more studies are needed to evaluate the clinical usability of GDF‐15 as a marker of frailty or sarcopenia and to investigate the prognostic value of GDF‐15 in terms of predicting both geriatric syndromes as well as adverse outcomes related to older age, such as prolonged length of stay, decreased functional ability, and mortality. Notably, given the multicomponent concept of frailty and sarcopenia, it is highly likely that the predictive value of GDF‐15 increases when it is combined with several other markers in a predictive model.

## Conclusion

The present data demonstrate that plasma GDF‐15, a known biomarker associated with senescence and mitochondrial dysfunction, is associated with several geriatric conditions in acutely admitted older medical patients. Systemic GDF‐15 was higher in acutely admitted older patients with sarcopenia and frailty compared with patients without. Moreover, the present study defined the optimum cutoff for GDF‐15 related to the presence of sarcopenia and frailty, respectively. When elevated above the defined cutoffs, GDF‐15 was strongly associated with frailty and sarcopenia in both crude and fully adjusted models.

## Conflict of interest

The authors report no conflicts of interest.

## Funding

The work is supported by funding from the Novo Nordisk Foundation; grant number NNF18OC0052826.

## Supporting information


**Table S1.** Proportions in the different groups of the CCI weighted index and sex (for frailty alone) associated with GDF‐15 levels based on the optimum cut‐offs for frailty and sarcopenia, respectively.
