# Supplementary material for: GDF‐15 is associated with sarcopenia and frailty in acutely admitted older medical patients
Source: J Cachexia Sarcopenia Muscle. 2024 Jun 18;15(4):1549–57. doi: 10.1002/jcsm.13513 (PMC11294026; doi:10.1002/jcsm.13513)
Supplement: Supplementary file 1 — Table S1. Proportions in the different groups of the CCI weighted index and sex (for frailty alone) associated with GDF‐15 levels based on the optimum cut‐offs for frailty and sarcopenia, respectively. [file JCSM-15-1549-s001.docx]

**Table 3** Proportions in the different groups of the CCI weighted index and sex (for frailty alone) associated with GDF-15 levels based on the optimum cut-offs for frailty and sarcopenia, respectively.

|  | GDF-15  <1541 pg/ml | GDF-15  >1541 pg/ml | P-value |  | GDF-15  <2166 pg/ml | GDF-15  > 2166 pg/ml | P-value |
| --- | --- | --- | --- | --- | --- | --- | --- |
| CCI low n=25^a^ | 11 (44.0%) | 14  (56.0%) | P>0.4 | CCI low n=34 | 21  (61.8%) | 13  (38.2%) | P>0.1 |
| CCI moderate  n=264^a^ | 101 (38.3%) | 163  (61.7%) | **P<0.001^b^** | CCI moderate  n=400 | 176 (44.0%) | 224 (56.0%) | **P<0.02^b^** |
| CCI severe n=339^a^ | 81  (23.9%) | 258  (76.1%) | **P<0.001^b^** | CCI severe n=603 | 201 (33.4%) | 401  (66.6%) | **P<0.001^b^** |
|  | - | - | **-** | Male sex  n=488 | 152 (38.2%) | 336  (52.7%) | **<0.001^c^** |

^a^the proportions according to the GDF-15 cut-off related to sarcopenia are based on the subgroup of patients assessed for potential sarcopenia (n=628).^b^Chi-square test. ^c^Risk difference (95% CI): -0.14 (-0.20-0.08). Charlson Comorbidity Index (CCI); Confidence Interval (CI); Growth Differentiating Factor 15 (GDF-15)
